# Extreme weather-related knowledge level and educational needs among medical students: a multi-city cross-sectional study in eastern China

**DOI:** 10.3389/fpubh.2026.1891426

**Published:** 2026-07-27

**Authors:** Jian Cheng, Yuefang Li, Wenjun Cheng, Xiaojie Zou, Yarui Fan, Jian Song, Yinguang Fan

**Affiliations:** Department of Epidemiology and Biostatistics, School of Public Health, Anhui Medical University, Hefei, China

**Keywords:** climate change, educational needs, extreme weather, health, healthcare students

## Abstract

**Objective:**

Extreme weather remains a global health threat and has become more intense and frequent in the context of climate change. Mitigating the negative health impact of extreme weather events largely depends on efforts from medical and health workers, making the education on extreme weather-related knowledge among healthcare students paramount importance. However, little is known about healthcare students’ knowledge level of extreme weather events and their educational needs. This study aimed to investigate the extent to which that healthcare students know about health impact of extreme weather events and their educational needs.

**Methods:**

A multi-city cross-sectional survey was conducted in eastern China from June to December in 2025. Data on basic characteristics, mastery of knowledge regarding climate change and extreme weather events (heatwave, coldwave, blizzard, and flood), educational experience and needs regarding extreme weather, and climate change anxiety were collected from healthcare students.

**Results:**

In general, healthcare students showed a low level of climate change anxiety, but their mastery of relevant knowledge was also low, with a relatively better mastery of heatwave-related knowledge and poorer mastery of coldwave-related knowledge. In addition, only a small proportion of students had course experience related to extreme weather (33.39%). Nevertheless, students expressed a strong willingness to learn (85.57%), with the desired learning content pertaining to the biological mechanisms by which extreme weather affects health, protection measures for high-risk populations under extreme weather conditions, campus emergency response, and methods to protect students’ mental health. Moreover, regarding course format preferences, most students favored flexible formats including elective courses (35.93%), thematic lectures (30.67%) and online courses (25.50%), while less students chose compulsory courses (5.90%) or other formats (2.00%).

**Conclusion:**

Healthcare students in eastern China have poor knowledge of extreme weather and health. In the context of ongoing global warming and students’ strong willingness to learn, medical curricula should be expanded to include education on extreme weather events and associated health impacts.

## 1 Introduction

Climate change is considered one of the greatest global health threats of the 21st century, with the frequency, intensity, and duration of extreme weather events continuing to increase (1). These extreme weather events can seriously affect multiple human organs and systems. For example, heatwave is associated with the increased risk of cardiovascular and respiratory diseases, while cold spell may elevate mortality risk among individuals with cognitive impairment (2, 3).

In response to the growing challenge posed by climate change, scholars worldwide have called for effective actions (4, 5). Previous studies have shown that knowledge is an important factor influencing climate-related behaviors and support for climate policies (6, 7). Consequently, integrating climate change–related content into educational system has been increasingly recognized as a key strategy for improving public awareness and adaptive capacity (8, 9). A recent survey published by The Lancet reported that, during 2023–2024, 62 of 81 countries had implemented education on climate change and health (10). This suggests that many countries have begun incorporating climate change and health education into school curricula as part of broader climate-related governance efforts.

Healthcare professionals, including physicians, nurses, public health practitioners, and health officials, play a critical role in reducing the health risk of climate change (11, 12). In addition to identifying and managing extreme weather-related health conditions, they can also promote public health education and sustainable practices because they are generally trusted by the public (13, 14). As future members of the healthcare workforce, healthcare students’ awareness of and capacity to respond to the health risk associated with extreme weather events will directly influence future clinical and public health practice (15, 16). Therefore, assessing healthcare students’ knowledge of extreme weather–related health risk and their educational needs is essential for identifying gaps in medical education and strengthening the preparedness of future healthcare professionals (17).

However, existing studies remain limited and have mainly focused on specific subgroups of healthcare students, such as nursing students (18, 19). Educational needs regarding extreme weather-related knowledge among the broader medical student population remain largely unclear. Moreover, previous research has identified differences within medical student populations in their understanding of the relationship between climate change and health, yet the sources and distribution of these differences have not been fully explored (20, 21). Therefore, this multi-city cross-sectional study in Anhui Province of eastern China had two objectives: (1) to assess healthcare students’ knowledge of climate change and the health impact of extreme weather events using a structured questionnaire with predefined scoring criteria; and (2) to evaluate their educational needs regarding extreme weather–related health content. We also examined differences in knowledge levels and educational needs across genders, grades, and majors.

## 2 Methods

### 2.1 Study design and setting

From June to December 2025, a multi-region cross-sectional survey was conducted at five universities in eastern China, which enroll healthcare students across China. Specifically, this survey covered three regions of Anhui Province: southern Anhui, central Anhui, and northern Anhui. Five universities are Anhui Medical University and the Clinical Medical College of Anhui Medical University in Hefei city (central Anhui), Bengbu Medical University in Bengbu and Anhui University of Science and Technology in Huainan city (northern Anhui), as well as Wannan Medical University in Wuhu city (southern Anhui).

### 2.2 Sample size

The required sample size was estimated using the standard formula for the cross-sectional study (22).

$$N=\frac{Z_{\alpha }^{2}\times p\times (1-p)}{d^{2}}$$

Where N represents the required sample size, $Z_{\alpha }^{2}$ represents the standard normal value corresponding to the selected confidence level, p denotes the anticipated proportion of the main survey indicator, and d represents the allowable margin of error. In this study, a 95% confidence level was adopted, corresponding to $Z_{\alpha }^{2}$ =1.96. Based on a previous study among medical students, 55.5% of healthcare students correctly answered questions related to health co-benefits (20). Therefore, p was set as 0.555. The allowable margin of error was defined as 10% of p , namely d = 0.0555. Using these parameters, the minimum required sample size was calculated to be 309. To ensure an adequate sample size and account for potential invalid or incomplete questionnaires, the sample size was increased by 10%, resulting in a final minimum sample size of 340.

### 2.3 Participants

This study included healthcare students from various medical disciplines, such as public health, clinical medicine and others. In addition, all participants meet the following inclusion criteria: (a) undergraduate students, (b) healthcare students, and (c) voluntary participation.

### 2.4 Questionnaire development

The self-designed items included: (i) students’ basic information, such as gender, grade, and major; (ii) students’ knowledge of four common extreme events (heat waves, cold waves, blizzards, and floods); and (iii) students’ previous course experience, future learning willingness, and educational needs on relevant knowledge. The five knowledge-related items were developed mainly based on relevant standards and regulations issued by the China Meteorological Administration, including the Grade of Cold Air (GB/T 20484-2017), the Grade of Precipitation (GB/T 28592-2012), and the Measures for the Issuance and Dissemination of Meteorological Disaster Warning Signals (23–25). Then, the remaining self-designed items were developed with reference to previous studies (9, 10, 26–28). Knowledge scores ranged from 0 to 5 (1 point per correct answer) and were categorized as low (≤3) or high (≥4) (29).

In addition, we used the Climate Change Anxiety Scale (CCAS), a validated instrument in Chinese populations, to assess students’ climate change anxiety (30–32). It consists of 13 items rated on a 5-point Likert scale, yielding a total score ranging from 13 to 65, with the higher score indicating a higher level of climate change anxiety (33, 34). Based on a previous study, participants with CCAS scores < 28 and ≥28 were classified as low- and high-risk groups, respectively (35). Furthermore, internal consistency was assessed using Cronbach’s alpha, indicating excellent reliability (Cronbach’s *α* = 0.96). In addition, confirmatory factor analysis (CFA) was conducted to examine whether the previously reported factor structure of the CCAS was applicable to the current sample (36). The CFA yielded the following fit indices: CFI = 0.90, TLI = 0.88, RMSEA = 0.14, and SRMR = 0.05.

The self-designed items were reviewed by several experts in environmental epidemiology and revised according to their comments. The survey was then pilot-tested among 25 postgraduate students to assess the overall completion process and clarity of instructions.

### 2.5 Data collection

Prior to the formal survey, we provided detailed explanations for each question. All participants completed the survey via the professional online survey platform namely Wenjuanxing^1^ that has been widely used in previous studies of China (37–39). Additionally, to ensure the integrity and quality of the data, the survey included attention check questions (e.g.,“Please select the answer ‘Blue’”) (40). These restrictions effectively prevented information bias caused by incorrect responses and passive participation.

### 2.6 Statistical analysis

Descriptive statistics were used to summarize the characteristics of the participants. Continuous variables were presented as mean ± standard deviation (SD) or median (interquartile range, IQR) as appropriate based on data distribution, whereas categorical variables were summarized as frequencies and percentages. For comparisons between groups, categorical variables were analyzed using the chi-square test or Fisher’s exact test. All tests were two-sided, and a *p < 0.05* was considered statistically significant. All statistical analyses in this study were conducted using the “dplyr,” “tidyr,” “stats,” “nortest” and “ggplot2” packages in R software (version: 4.4.1).

### 2.7 Ethical approval and informed consent

At the beginning of the questionnaire, all participants were informed of the study’s purpose, procedures, their rights, and that participation was voluntary. Completion of the questionnaire was considered to indicate informed consent to participate. This study was approved by the Ethics Committee of Anhui Medical University (Approval No. 81250722).

## 3 Results

### 3.1 Characteristics of participants

A total of 1,102 valid questionnaires out of 1,200 questionnaires (validation rate: 91.83%) were used for final data analysis (Supplementary Figure S1). Among the 98 excluded questionnaires, 16 were incomplete, 13 lacked basic information, 31 were excluded for too-fast completion (<164 s, below the 5th percentile), and 38 failed the attention check. Participant characteristics are presented in Table 1. Of the 1,102 respondents, 33.12% were males and 66.88% were females. Participants were predominantly from second-year (45.74%) and third-year (24.59%) students, followed by first-year (14.88%) and fourth-year or above (14.79%). In terms of majors, public health accounted for 36.7% and clinical medicine accounted for 22.6%, with the remaining 40.7% from other majors. Most participants came from central (40.9%) and northern Anhui (37.6%), with southern Anhui comprising 21.5%. Overall, 93.2% of students reported experiencing at least one extreme weather event in the past 3 years, whereas 6.8% reported no such experience. In the total study population, heatwave was the most frequently reported event (32.7%), followed by coldwave (24.7%), flood (19.9%), and blizzard (16.0%).

**Table 1:** General characteristics of healthcare students in Anhui province (*N* = 1,102).

Characters		*N*	%	Characters		*N*	%
Gender	Male	365	33.12	Experience^a^	Heatwave		
	Female	737	66.88		Yes	360	32.67
Grade	First-year	164	14.88		No	742	67.33
	Second-year	504	45.74		Coldwave		
	Third-year	271	24.59		Yes	272	24.68
	Fourth-year and above	163	14.79		No	830	75.32
Major	Public health	404	36.66		Blizzard		
	Clinical medicine	249	22.6		Yes	176	15.97
	Others	449	40.74		No	926	84.03
Region	Central	451	40.93		Flood		
	Northern	414	37.57		Yes	219	19.87
	Southern	237	21.51		No	883	80.13

a Extreme weather events experienced in hometown in the past 3 year.

### 3.2 Students’ knowledge, climate anxiety, and educational needs related to extreme weather

**Figure 1:**
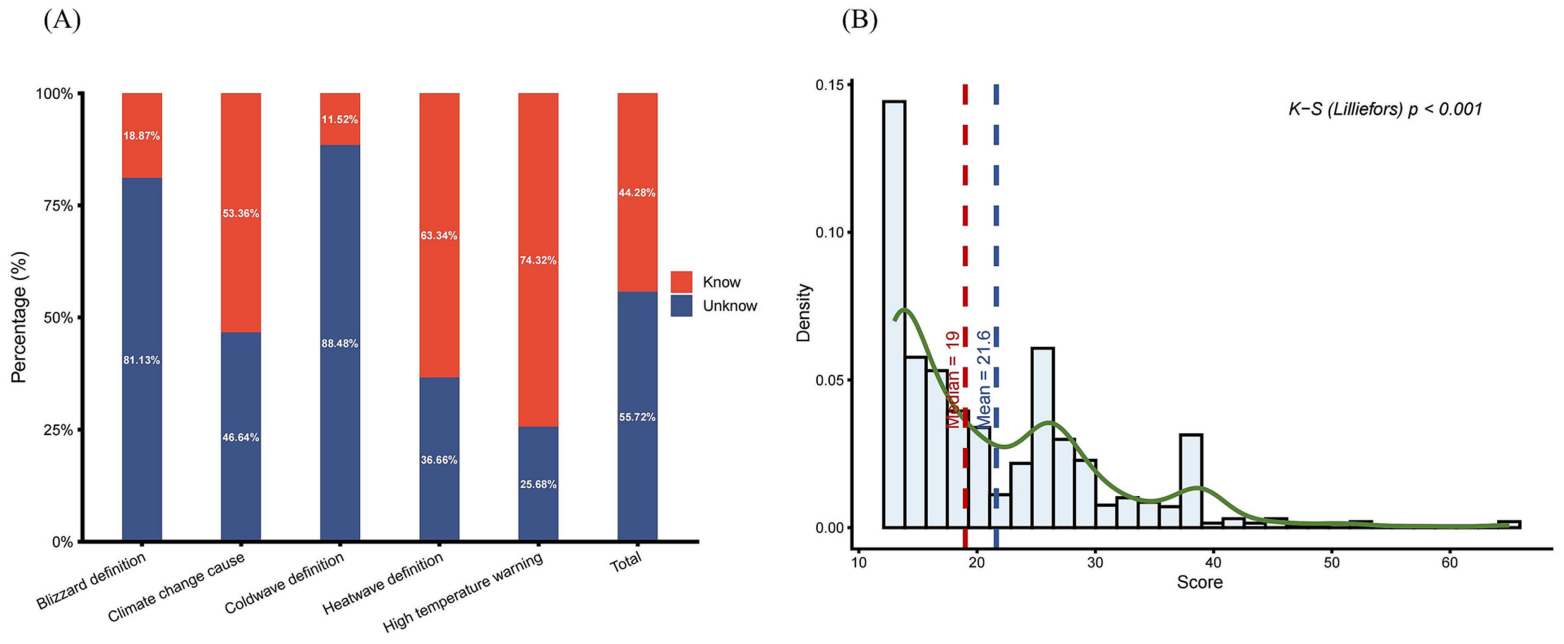
Healthcare students’ knowledge level of extreme weather **(A)** and anxiety levels about climate change **(B)**. K-S (Lilliefors): Lilliefors-corrected Kolmogorov–Smirnov. The K-S (Lilliefors) *p* < 0.001 indicates the distribution is non-normal. A higher mean value than the median value indicates the distribution is right-skewed. CCAS: Climate Change Anxiety Scale.

We observed a relatively low level of knowledge related to extreme weather in total, only reaching 44.28% (Figure 1A). Specifically, healthcare students demonstrated a low level of knowledge regarding the definitions of coldwave and blizzard, only reaching 11.52 and 18.87%, respectively. However, they demonstrated a better grasp of high-temperature warning, the definition of heatwave, and the primary causes of climate change, reaching 74.32, 63.34, and 53.36%, respectively. Furthermore, CCAS scores were positively skewed, with a median of 19 (P25-P75: 13–26) (Figure 1B). Most healthcare students exhibited a low level of climate change anxiety (79.13%), while 20.87% showed a high level.

**Figure 2:**
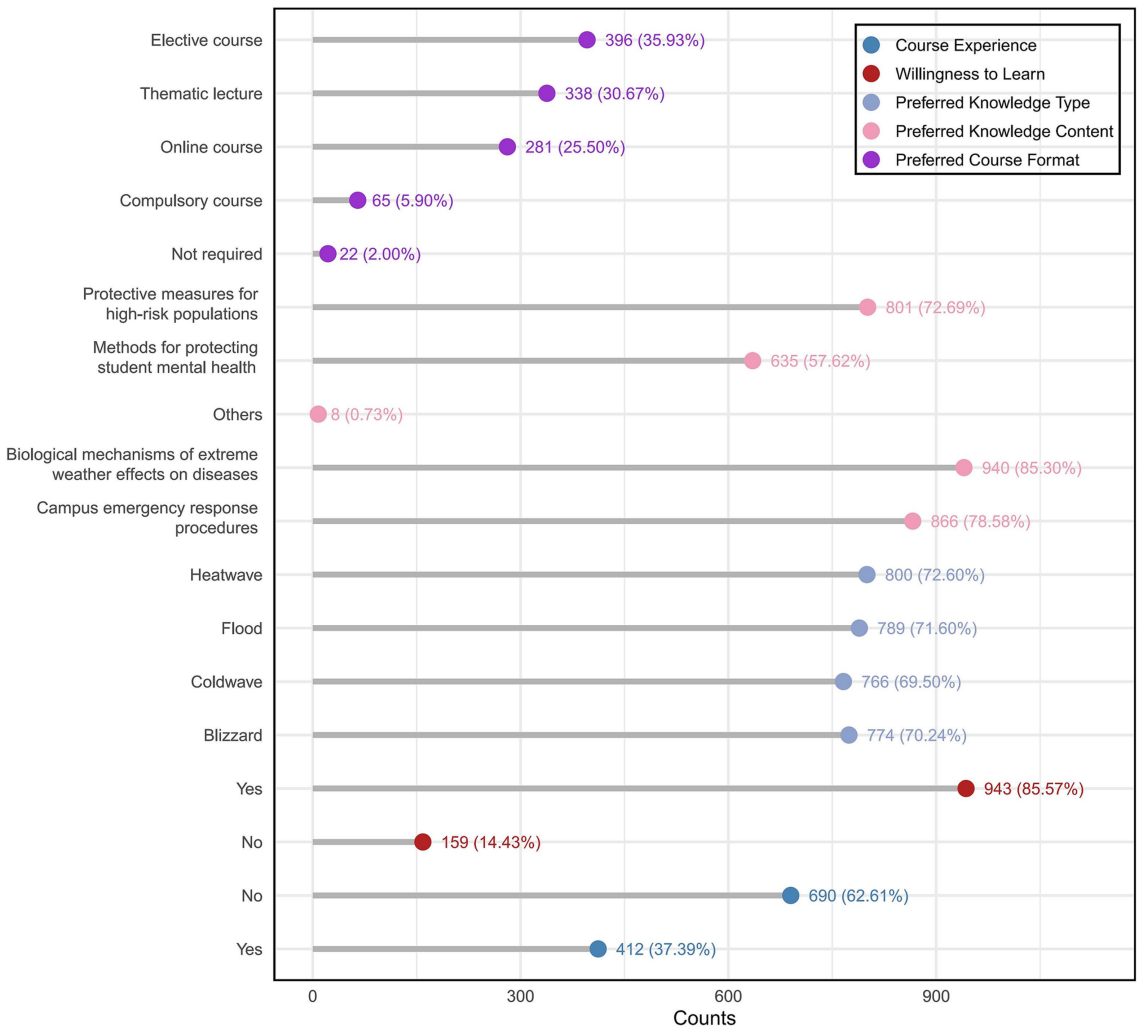
Healthcare students’ learning willingness and educational needs regarding extreme weather events.

Regarding extreme weather-related course experience, willingness to learn, and educational needs (i.e., knowledge type, knowledge content, and course format) (Figure 2). Only 37.39% of students reported prior course exposure to relevant courses, while 62.61% had not received such education. However, 85.57% of students expressed willingness to learn about climate change-related content in the future. For knowledge type (multiple-response), students’ interests were relatively balanced across extreme weather categories, including heatwave (72.60%), flood (71.60%), blizzard (70.24%), and coldwave (69.50%). Regarding knowledge content (multiple-response), the most frequently reported need was biological mechanisms of extreme weather effects on diseases (85.30%), followed by campus emergency response procedures (78.58%), protective measures for high-risk populations under extreme weather (72.69%), and methods for protecting students’ mental health (57.62%). As for course format (single-choice), elective course (35.93%) was the most preferred option, followed by thematic lectures (30.67%) and online courses (25.50%), while fewer students selected compulsory courses (5.90%) or indicated that the course was not necessary (2.00%).

### 3.3 Differences of course experience, learning willingness, and educational needs by student characteristics

Regarding course experience and willingness to learn (Table 2), no significant difference in course experience was observed across gender or major groups (*p > 0.05*). However, course experience varied significantly by grade, with first-year students reporting the highest proportion of prior course and students in the fourth year and above reporting the lowest proportion (first-year 6.62% vs. second-year 16.61% vs. third-year 9.71% vs. fourth-year and above 4.44%, *p = 0.04*). In terms of willingness to learn, significant differences were observed by gender and grade. Female students demonstrated a higher willingness to learn compared with male students (59.17% vs. 26.41%, *p < 0.01*). Differences were also found across grades, with first-year students showing the lowest proportion (13.97%), followed by third-year (32.76%) and second-year students (38.84%), while fourth-year and above students accounted for 11.52% (*p = 0.04*). In contrast, no significant differences were observed across different majors (*p = 0.48*).

**Table 2:** Comparison of extreme weather-related course experience and willingness to learn relevant knowledge by general characteristics.

Characters		Course experience		Willingness to learn	
		Yes, *n* (%)	No, *n* (%)	Yes, *n* (%)	No, *n* (%)
Gender	Male	140 (12.70%)	225 (20.42%)	291 (26.41%)	74 (6.72%)
	Female	272 (24.68%)	465 (42.20%)	652 (59.17%)	85 (7.7%)
	*χ* ^2^	0.16		14.41	
	*p*	0.69		**<0.01**	
Grade	First-year	73 (6.62%)	91 (8.25%)	154 (13.97%)	10 (0.90%)
	Second-year	183 (16.61%)	321 (29.13%)	428 (38.84%)	76 (6.90%)
	Third-year	107 (9.71%)	164 (14.88%)	361 (32.76%)	73 (6.62%)
	Fourth-year and above	49 (4.44%)	114 (10.34%)	127 (11.52%)	36 (3.27%)
	*χ* ^2^	8.05		17.27	
	*p*	**0.04**		**<0.01**	
Major	Clinical medicine	154 (13.97%)	250 (22.67%)	345 (31.31%)	59 (5.35%)
	Others	153 (13.88%)	296 (26.86%)	390 (35.40%)	59 (5.35%)
	Public health	105 (9.52%)	144 (13.07%)	208 (18.87%)	41 (3.72%)
	*χ* ^2^	4.63		1.45	
	*p*	0.10		0.48	

Bold values indicate statistical significance (*P* < 0.05).

Regarding educational needs (Table 3), significant gender differences were observed in preferences for heatwave (*p = 0.02*), flood (*p = 0.03*), and blizzard-related knowledge (*p < 0.01*), with female students generally showing higher educational needs. No significant grade difference was identified in preferred knowledge types (*p > 0.05*). Across majors, significant difference was only observed for flood-related knowledge (*p < 0.01*), with public health students reporting a less demand.

**Table 3:** Educational needs on extreme weather among healthcare students by different characteristics.

		Preferred knowledge type, *n* (%)								Preferred knowledge content, *n* (%)								Preferred course format, *n* (%)				
		Heatwave		Coldwave		Flood		Blizzard		Biological mechanisms of extreme weather effects on diseases		Protective measures for high-risk populations		Campus emergency response procedures		Methods for protecting student mental health		Compulsory course	Elective course	Thematic lecture	Online course	Not required
		Yes	No	Yes	No	Yes	No	Yes	No	Yes	No	Yes	No	Yes	No	Yes	No	Yes	Yes	Yes	Yes	Yes
Gender	Male	249 (22.59%)	116 (10.53%)	246 (22.32%)	119 (10.80%)	246 (22.32%)	119 (10.80%)	235 (21.32%)	130 (11.80%)	305 (27.68%)	60 (5.44%)	241 (21.87%)	124 (11.25%)	258 (23.41%)	107 (9.71%)	195 (17.70%)	170 (15.43%)	28 (2.54%)	138 (12.52%)	97 (8.80%)	86 (7.80%)	16 (1.45%)
	Female	551 (50.00%)	186 (16.88%)	520 (47.2%)	217 (19.69%)	543 (49.27%)	194 (17.60%)	539 (48.91%)	198 (17.97%)	635 (57.62%)	102 (9.26%)	560 (50.82%)	177 (16.06%)	608 (55.17%)	129 (11.71%)	440 (39.93%)	297 (26.95%)	37 (3.36%)	258 (23.41%)	241 (21.87%)	195 (17.70%)	6 (0.54%)
	*p*	**0.02**		0.28		**0.03**		**<0.01**		0.25		**<0.01**		**<0.01**		0.05		**0.02**				
Grade	First-year	125 (11.34%)	39 (3.53%)	111 (10.1%)	53 (4.81%)	129 (11.71%)	35 (3.18%)	122 (11.07%)	42 (3.81%)	149 (13.52%)	15 (1.36%)	130 (11.80%)	34 (3.09%)	140 (12.70%)	24 (2.18%)	114 (10.34%)	50 (4.54%)	15 (1.36%)	28 (2.54%)	76 (6.90%)	45 (4.08%)	0 (0.0%)
	Second-year	365 (33.12%)	139 (12.61%)	354 (32.1%)	150 (13.61%)	361 (32.76%)	143 (12.98%)	347 (31.49%)	157 (14.25%)	429 (38.93%)	75 (6.81%)	346 (31.40%)	158 (14.34%)	385 (34.94%)	119 (10.80%)	278 (25.23%)	226 (20.51%)	25 (2.27%)	183 (16.61%)	140 (12.70%)	142 (12.89%)	14 (1.27%)
	Third-year	199 (18.10%)	72 (6.53%)	189 (17.2%)	82 (7.44%)	187 (16.97%)	84 (7.62%)	197 (17.88%)	74 (6.72%)	232 (21.05%)	39 (3.54%)	206 (18.70%)	65 (5.90%)	223 (20.24%)	48 (4.36%)	160 (14.52%)	111 (10.07%)	15 (1.36%)	116 (10.53%)	74 (6.72%)	63 (5.72%)	3 (0.28%)
	Fourth-year and above	111 (10.07%)	52 (4.72%)	112 (10.2%)	51 (4.63%)	112 (10.16%)	51 (4.63%)	108 (9.80%)	55 (5.00%)	130 (11.80%)	33 (2.99%)	119 (10.80%)	44 (4.00%)	118 (10.71%)	45 (4.08%)	83 (7.53%)	80 (7.26%)	10 (0.91%)	69 (6.26%)	48 (4.36%)	31 (2.81%)	5 (0.45%)
	*p*	0.42		0.92		0.13		0.28		**0.04**		**0.03**		**0.01**		**<0.01**		**<0.01**				
Major	Clinical medicine	297 (26.95%)	107 (9.71%)	277 (25.1%)	127 (11.52%)	293 (26.59%)	111 (10.07%)	279 (25.32%)	125 (11.34%)	341 (30.94%)	63 (5.72%)	308 (27.95%)	96 (8.71%)	314 (28.49%)	90 (8.17%)	247 (22.41%)	157 (14.25%)	32 (2.90%)	151 (13.70%)	129 (11.71%)	88 (7.99%)	4 (0.36%)
	Others	328 (29.76%)	121 (10.98%)	313 (28.4%)	136 (12.34%)	340 (30.86%)	109 (9.89%)	326 (29.58%)	123 (11.16%)	388 (35.21%)	61 (5.53%)	310 (28.31%)	139 (12.61%)	363 (32.94%)	86 (7.80%)	250 (20.69%)	199 (18.06%)	24 (2.18%)	143 (12.98%)	133 (12.07%)	138 (12.52%)	11 (1.00%)
	Public health	175 (15.88%)	74 (6.71%)	176 (16.0%)	73 (6.62%)	156 (14.16%)	93 (8.44%)	169 (15.34%)	80 (7.26%)	211 (19.15%)	38 (3.45%)	183 (16.61%)	66 (5.99%)	189 (17.15%)	60 (5.44%)	138 (12.52%)	111 (10.07%)	9 (0.82%)	102 (9.26%)	76 (6.90%)	55 (5.00%)	7 (0.64%)
	*p*	0.64		0.84		**<0.01**		0.34		0.68		0.06		0.27		0.20		**0.01**				

Bold values indicate statistical significance (*P* < 0.05).

For preferred knowledge content, grade differences were observed in biological mechanisms of extreme weather effects on diseases (*p = 0.04*), protective measures for high-risk populations (*p = 0.03*), campus emergency response procedures (*p = 0.01*), and methods for protecting students’ mental health (*p < 0.01*). Female students also reported greater demand for protective measures for high-risk populations and campus emergency response procedures (*p < 0.01*). No significant differences in preferred knowledge contents were observed across majors (*p > 0.05*).

In addition, preferences for course format differed significantly by gender (*p = 0.02*), grade (*p < 0.01*), and major (*p = 0.01*). Overall, elective courses, thematic lectures and online course were the most preferred formats among students.

## 4 Discussion

This multi-city study in eastern China found that healthcare students demonstrated relatively low extreme weather-related knowledge level, with variations in distinct extreme weather events. For instance, students demonstrated better knowledge of extreme high-temperature events than of coldwave and blizzard events. This finding is consistent with previous studies across Asia, likely reflecting the increasing visibility and public health burden of heat extremes in the region (41, 42). One possible explanation is that high temperature events have become more frequently in recent years in the studied region, and related warning information has been widely disseminated, which may have facilitated students’ acquisition of relevant knowledge through multiple information sources (43). These findings suggest that future consideration is warranted for incorporating training on extreme weather-related health knowledge into medical education, particularly low-temperature events such as coldwave and blizzard.

Although the overall level of climate change anxiety among healthcare students was relatively low, 20.87% of participants were classified as having high climate anxiety, indicating that a non-negligible subgroup may experience elevated emotional burden related to climate change. Previous studies have suggested that excessive climate change anxiety may increase psychological stress and negatively affect learning and daily life (44, 45). Therefore, these findings further highlight the importance and necessity of integrating climate change-related mental health content into medical education. Relevant curriculum content may include the mental health impact of climate change, psychological coping strategies, and recognition of climate-related emotional distress.

Regarding willingness to learn, our findings were consistent with previous studies showing that females tend to have greater concern about climate change (45, 46). In our survey, we observed significantly higher learning willingness among female students. In addition, lower-grade students exhibited stronger learning willingness than higher-grade students, which may be attributed to the increased academic, graduation, and employment pressure faced by senior students, potentially reducing their willingness to engage in the learning (47).

With respect to the educational needs, there were differences in the demand for extreme weather-related knowledge among students of different genders, grades, and majors. Female students showed higher demand for multiple types of knowledge, as well as for content on high-risk population protection and campus emergency response, which is consistent with previous findings that women pay more attention to the impact of climate change (20, 46). Differences were also observed across majors. In particular, public health students reported greater demand for flood-related knowledge and showed stronger interest in the biological mechanisms of extreme weather effects on diseases, protective measures, and methods for protecting students’ mental health; this finding indicates that difference in academic background may lead to variations in students’ understanding and mastery of relevant knowledge.

In terms of course formats, students generally preferred non-compulsory courses. Notably, student preferences do not necessarily reflect teaching effectiveness (48). Therefore, course design should balance students’ acceptance with educational objectives, and carefully consider both the effectiveness and feasibility of different instructional formats. In this context, novel approaches combining lectures, case-based discussions, simulation exercises, and community-based practice may provide more comprehensive training and improve both engagement and learning outcomes.

This study has two advantages. First, it is a multi-center survey including healthcare students from different majors and grades, which enhanced the representativeness and reliability of the findings. Second, the results may inform the integration of climate change and extreme weather-related knowledge into medical education.

However, limitations of this study should be acknowledged as well. Firstly, this study was conducted only in Anhui Province, and the sample included a higher proportion of female and lower-grade students, which may limit generalizability to other regions. Secondly, the cross-sectional study design suggests that the recall bias exist when answering the questions in the survey, which to some extent have affected the final results. Thirdly, multiple-comparison corrections, such as the Bonferroni, were not applied, which may increase the risk of type I error. Finally, all analyses were based on unadjusted comparisons, and potential confounding factors such as gender, major, and grade were not controlled for, which may affect subgroup interpretations. Future studies should apply multivariable analytical approaches to better control for confounding factors and strengthen inference. In addition, educational interventions should be evaluated through pilot implementation in selected medical schools followed by longitudinal studies to assess their effectiveness in improving students’ knowledge, preparedness, and psychological adaptation.

## 5 Conclusion

Overall, this study suggests that healthcare students in eastern China demonstrate a relatively low level of knowledge regarding extreme weather-related health risks, but a high willingness to learn relevant knowledge, particularly among the first-year students and female students. Given the substantial health risks posed by extreme weather and the associated burden on healthcare systems, these findings support integrating targeted education on extreme weather-related health effects into medical curricula. Priority may be given to topics with a lower knowledge level, such as coldwave and blizzard, while adopting instructional formats are both educationally effective and acceptable to healthcare students. These findings should be interpreted within the context of a single-province, cross-sectional study among healthcare students in eastern China, and may not be fully generalizable to students in other regions.

## Data Availability

The datasets presented in this article are not readily available because the authors do not have permission to share data of this study. Requests to access the datasets should be directed to Jian Cheng, jiancheng@ahmu.edu.cn.

## Footnotes

1
https://www.wjx.cn/app/survey.aspx
