# Supplementary material for: Extreme weather-related knowledge level and educational needs among medical students: a multi-city cross-sectional study in eastern China
Source: Front Public Health. 2026 Jul 27;14:1891426. doi: 10.3389/fpubh.2026.1891426 (PMC13453731; doi:10.3389/fpubh.2026.1891426)
Supplement: Supplementary file 1 [file Data_Sheet_1.docx]

**SUPPLEMENTAL MATERIAL**

**Extreme weather-related knowledge level and educational needs among medical students: A multi-city cross-sectional study in eastern China**

Jian Cheng^a,1,^*, Yuefang Li^a,1^, Wenjun Cheng^a^, Xiaojie Zou^a^, Yarui Fan^a^, Jian Song^a^, Yinguang Fan^a^

a Department of Epidemiology and Biostatistics, School of Public Health, Anhui Medical University, Hefei, China;

1 Co-first authors (these two authors contributed equally to this work);

* Corresponding author: Jian Cheng, School of Public Health, Department of Epidemiology and Biostatistics, Anhui Medical University, 81 Meishan Road, 230032, Hefei, China (e-mail: jiancheng@ahmu.edu.cn).

| **Content** | **Page** |
| --- | --- |
| **Fig. S1** Flowchart of questionnaire screening and sample selection. | **3** |

**Fig. S1** Flowchart of questionnaire screening and sample selection.
